# Targeting Hepatic Protein Carbonylation and Oxidative Stress Occurring on Diet-Induced Metabolic Diseases through the Supplementation with Fish Oils

**DOI:** 10.3390/md16100353

**Published:** 2018-09-26

**Authors:** Silvia Muñoz, Lucía Méndez, Gabriel Dasilva, Josep Lluís Torres, Sara Ramos-Romero, Marta Romeu, María Rosa Nogués, Isabel Medina

**Affiliations:** 1 Instituto de Investigaciones Marinas, Consejo Superior de Investigaciones Científicas (IIM-CSIC), E-36208 Vigo, Spain; luciamendez@iim.csic.es (L.M.); gabrieldasilvaalonso@gmail.com (G.D.); medina@iim.csic.es (I.M.); 2 Instituto de Química Avanzada de Catalunya, Consejo Superior de Investigaciones Científicas (IQAC-CSIC) Jordi Girona 18-26, E-08034 Barcelona, Spain; joseplluis.torres@iqac.csic.es (J.L.T.); sara.ramosromero@gmail.com (S.R.-R.); 3 Unidad de Farmacología, Facultad de Medicina, Universidad Rovira i Virgili, Sant Llorenç 21, E-43201 Reus, Spain; marta.romeu@urv.cat (M.R.); mariarosa.nogues@urv.cat (M.R.N.)

**Keywords:** oxidative stress, high-fat high-sucrose diet, liver protein damage, marine omega-3 fatty acids, carbonylation, fish oils, Sprague-Dawley rat

## Abstract

The present study addressed the ability of long-chain ω-3 polyunsaturated fatty acids (ω-3 PUFA), i.e., eicosapentaenoic acid (EPA) and docosahexaenoic acid (DHA), to ameliorate liver protein damage derived from oxidative stress and induced by consumption of high-caloric diets, typical of Westernized countries. The experimental design included an animal model of Sprague-Dawley rats fed high-fat high-sucrose (HFHS) diet supplemented with ω-3 EPA and DHA for a complete hepatic proteome analysis to map carbonylated proteins involved in specific metabolic pathways. Results showed that the intake of marine ω-3 PUFA through diet significantly decreased liver protein carbonylation caused by long-term HFHS consumption and increased antioxidant system. Fish oil modulated the carbonylation level of more than twenty liver proteins involved in critical metabolic pathways, including lipid metabolism (e.g., albumin), carbohydrate metabolism (e.g., pyruvate carboxylase), detoxification process (e.g., aldehyde dehydrogenase 2), urea cycle (e.g., carbamoyl-phosphate synthase), cytoskeleton dynamics (e.g., actin), or response to oxidative stress (e.g., catalase) among others, which might be under the control of diet marine ω-3 PUFA. In parallel, fish oil significantly changed the liver fatty acid profile given by the HFHS diet, resulting in a more anti-inflammatory phenotype. In conclusion, the present study highlights the significance of marine ω-3 PUFA intake for the health of rats fed a Westernized diet by describing several key metabolic pathways which are protected in liver.

## 1. Introduction

The development of metabolic disorders such as obesity, type 2 diabetes and cardiovascular diseases are associated with the consumption of Westernized diets with an excess caloric intake of fat and sugar [1]. Oxidative stress plays a crucial role in the pathogenesis of these diet-related diseases. In general terms, the overproduction of reactive oxygen species (ROS) damage biological molecules altering cell function and leading to cell death. However, oxidative stress contributes to the development of these pathologies through multifactorial mechanisms, which are not fully understood yet [2]. For instance, prolonged exposition to high glucose concentration and fatty acids leads to an excess generation of ROS. Particularly, the superoxide anion overproduction by the mitochondria activate metabolic pathways involved in the development of diabetes complications, such as the increased formation of advanced glycation end-products (AGEs) and expression of their receptor and activating ligands [3,4,5]

The liver has been described as a major organ attacked by ROS [6]. Prolonged imbalance between the production of free radicals and their elimination by liver antioxidant defense system leads to extensive potential damage. The oxidative stress not only triggers hepatic damage by inducing irretrievable alteration of cell lipids but also proteins and DNA, affecting metabolic pathways that control normal biological functions [7]. Protein carbonyls represent an irreversible post-translational modification formed early during oxidative stress conditions. For this reason, it is the most general and well-used biomarker of severe oxidative protein damage [8]. The identification of a pattern for liver molecular alterations present at early stages of oxidative damage would be of great help in its early recognition and to monitor metabolic diseases evolution. 

Recent findings have shown that obesity induced by high-fat diets is accompanied by carbonylation increment of liver-regulatory proteins, thus suggests a link between diet, oxidative stress and metabolic disorders [9]. Furthermore, protein carbonylation is not only regulated by the amount of dietary fat but also by the type of fat. Therefore, the fatty acid composition of the diet rather than the amount of fat content per se may be a factor on the progression of metabolic diseases.

The latest advances in the identification of carbonylated proteins have demonstrated selective carbonylation of specific protein targets in muscle, heart and liver of rats fed with high-fat high-sucrose (HFHS) diets [10]. These studies should provide essential information in effectively understanding the metabolic disease process and the proper antioxidant therapy 

Many studies have reported that bioactive compounds such as polyphenols, vitamins and minerals prevent, or at least attenuate, oxidative stress derived injuries. Among them, marine ω-3 PUFA have been shown to modulate oxidative stress through reduction of protein oxidation and symptoms of metabolic disorders [11]. Previous results revealed that rats fed diets enriched with fish oil showed lower global carbonylation level in several body tissues than rats fed diets enriched with soybean or linseed oil. Fish oil also selectively modulated the carbonylation of body proteins [11]. Interestingly, the effect on protein carbonylation was closely dependent on the proportion of eicosapentaenoic acid (EPA) and docosahexaenoic acid (DHA), being the EPA and DHA ratio 1:1 the most effective one. 

Additionally, lipidomic approaches [12] have demonstrated that HFHS diets supplemented with fish oil to enhanced the activity of specific antioxidant enzymes related to oxidative stress as glutathione peroxidase (GPx) and modulated the synthesis of arachidonic acid (ARA) pro-inflammatory lipid mediators through the cyclooxygenase (COX) pathways. Therefore, the study of how ω-3 PUFA regulate oxidative stress preventing protein oxidation and its deleterious metabolic effects, can significantly contribute to understand the mechanisms underlying beneficial effects of marine lipids on metabolic health. 

Therefore, the present study aimed to examine the potential effects of ω-3 EPA and DHA from fish oil on the oxidative stress occurred in the liver of rats fed a high-caloric diet. To this purpose, we analyzed total and individual liver protein carbonylation of rats fed a HFHS diet for 24 weeks, compared rats fed HFHS supplemented with EPA and DHA in a ratio of 1:1. Rats fed a standard (STD) diet were used as a healthy control. The effect of fish oil on the hepatic carbonylation protein profile was correlated with biometrical and biochemical measurements as well as other oxidative stress and inflammation data to effectively gain knowledge in its beneficial metabolic role.

## 2. Results

### 2.1. Biometrical Parameters and Metabolic Plasma Values

Animals fed both HFHS diets showed a trend to increase body weight and perigonadal adipose tissue (Table 1). The consumption of HFHS diets significantly increased (almost 50%) the adiposity index related to STD diet. HFHS diet provoked a significant increment of plasma insulin (Table 1). Indeed, rats fed HFHS diets reached almost twice the level of their plasma insulin as compared with STD-fed rats. HFHS and HFHS + ω3 showed none significant differences in those parameters.

Other parameters such as plasma glucose, total cholesterol, low-density lipoprotein cholesterol (LDL) and high-density lipoprotein cholesterol (HDL) cholesterol, and triglycerides did not show any significant changes among the three groups. As regards to liver parameters, liver weight, liver fat content and the hepatosomatic index did not significantly change among the three groups either. The consumption of the HFHS diet did not increase enzymatic activities in plasma of alanine aminotransferase (ALT) and aspartate aminotransferase (AST), and even decreased them. However, the supplementation of the HFHS diet with fish oil rendered no significant differences in the ALT activity as compared to STD-fed rats. Altogether, the liver parameters indicated no fatty acid development after the intake of HFHS diets.

### 2.2. Liver Antioxidant Enzymes

Antioxidant enzymes in liver are shown in Table 2. HFHS diet presented significant lower enzymatic activity of most of the antioxidant enzymes (SOD, GPx and GR) as compared to STD diet. Lower amount of GSH and higher GSSG/GSH ratio were also found in these rats. The inclusion of EPA and DHA into the HFHS diet triggered significant higher activities of SOD, CAT, GPx and GR and more GSH content, yielding values not significantly different from STD diet.

### 2.3. Liver Fatty Acids Profile and Desaturases Indexes

Table S2 shows the fatty acid composition of each diet. It is important to point out that the fatty acids composition of marine lipids enriched feed had higher proportions of EPA, DPA, DHA, and ARA than control supplements (Table S2). Thereby, the fatty acids composition of STD and HFHS control diets had higher proportions of palmitic and oleic acids, linoleic acid (LA) and alfa-linolenic (ALA) acid than the HFHS diet supplemented with EPA and DHA. 

#### 2.3.1. TFA Incorporation into Liver

Liver corresponding to both dietary conditions clearly differed on the amount of 18:2ω6 (higher in standard feed), reflecting the different amounts of this fatty acid provided through the feeds (Table 3 and Table S2). Rats fed HFHS diet showed an excess of 18:1ω9 and 16:1ω7 in their liver in comparison with STD-fed rats. Both fatty acids are well-known markers of metabolic alterations [13]. The excess of the inflammatory markers oleic acid (18:1ω9) and palmitoleic acid (16:1ω7) found in HFHS livers decreased, although not significantly, in rats fed marine lipids. Similarly, there was a direct relationship between the accumulated levels of DHA and EPA in liver and the fish oil supplementation. However, the whole composition of fatty acids in the liver of animals did not reproduce exactly the composition of feeds. Livers from rats fed STD diet showed lower amount of 18:1ω9 and 18:2ω6 than their corresponding feeds and higher amounts of 16:0, 18:0 and ARA. Liver of animals fed HFHS decreased in the amount of 16:0 provided by diet, so as the level of 18:1ω9. Levels of 18:2ω6 were similar to the amount of fatty acid intake.

Supplementation with marine lipids largely increased the incorporation of ω-3 PUFA, EPA and DHA, in livers of rats fed HFHS diet and decreased the amounts of total ω-6 PUFA. This last finding could be mainly attributed to the decrease in the proportion of 20:4ω6 (ARA). Marine lipids incorporation modulated the level of other important fatty acids as well. Therefore, ω-3 PUFA supplementation showed a trend to decrease the amount of Linolenic acid, 18:3ω6 (ALA), in liver despite the amount of ARA and ALA coming from the HFHS feed. Consequently, when rats fed HFHS diets were supplemented with marine ω-3 PUFA, their livers decreased ARA amount and accumulated EPA and DHA increasing the sum of these two FA in 4.2%. As a general consequence, the inflammatory index ω-6/ω-3 in HFHS liver was significantly reduced after marine lipids treatments compared with HFHS control group (Table 3). 

Another important point is the selective incorporation into the liver found for DHA against EPA. Even if the ratio of the fatty acids in the supplemented fish oil was 1:1, results indicated an increase of 3.46% for DHA versus an increase of only 0.74% for EPA. This selective incorporation was also noticed in the increase of the ratio EPA:DHA after the supplementation with marine lipids. The proportion of EPA:DHA in liver was 0.068 and 0.048 in STD and HFHS groups, respectively (Table 3). The addition of marine lipids to the diet increases the ratio up to 0.10 in rats fed HFHS diets. Therefore, marine lipids supplementation was able to modulate liver fatty acid composition rather than total fat liver content.

#### 2.3.2. FADs Indexes

FAD indexes were calculated related to the liver total fatty acids. Results are shown in Table 4. The supplementation with marine ω-3 PUFA in HFHS diet tended to decrease both SCD-16 [palmitoleic (16:1ω7)/palmitic (16:0)] and SCD-18 [oleic (18:1ω9) /stearic (18:0)] and this finding could not be attributed to diet contribution. These trends were in agreement with the tendency to lower accumulation of the pro-inflammatory markers 16:1ω7 and 18:1ω9, as described above. 

Both HFHS diets intake resulted in higher values of Δ4D = [DHA (22:6ω3)/DPA (22:5ω3)] desaturase. However, there were no effects in Δ6D = [DGLA (20:3ω6)/LA (18:2ω6)] and Δ6D = [γ − ALA (18:3ω6)/LA (18:2ω6)] in accordance with the amounts of 20:3ω6 and 18:2ω6 provided by the feeds. 

On the other hand, no effects were observed after the supplementation of the HFHS diet with fish oils for Δ4D = [DHA (22:6ω3)/DPA (22:5ω3)], in accordance with the amounts of 22:6ω3 and 22:5ω3 provided by the feeds. None effects were observed for Δ6D = [DGLA (20:3ω6)/LA (18:2ω6)] and Δ6D = [γ − ALA (18:3ω6)/LA (18:2ω6)] either, which also agreed with the amounts of 20:3ω6 and 18:2ω6 provided by the feeds. However, fish oil significantly decreased Δ5D = [ARA (20:4ω6)/DGLA (20:3ω6)]. This result is in agreement with the amounts of 20:3ω6 and ARA incorporated through the marine lipid supplement to HFHS feeds. Since the ratio [20:4ω6/20:3ω6] in the control feeds (STD and HFHS) was higher than in the marine lipids supplemented feed, livers from rats belonging to control groups showed higher Δ5D desaturase values than livers corresponding to the marine group. Finally, significantly higher values of Δ5D/Δ6D = [EPA (20:5ω3)/ALA (18:3ω3)] desaturase were found in marine lipid supplemented rats according to fatty acids provided by feeds.

### 2.4. Lipid Peroxidation in Liver

Lipid peroxidation levels in liver were measured through conjugated dienes hydroperoxides (intermediate lipid oxidation product). Conjugated dienes hydroperoxides incremented in HFHS diet groups compared to STD group (Table 5). The amount of fat in liver was not affected by the supplementation of fish oils in animals fed HFHS, but the high level of liver lipid peroxidation that occurred showed a trend to be inhibited when ω-3 PUFA were provided.

The fat content of liver of STD and HFHS-fed rats was not statistically different. However, liver of rats fed HFHS showed a higher concentration of EPA and DHA than STD-fed animals according to the fatty acid composition. This means higher level of oxidizable substrate for the liver of animals fed HFHS than for animals fed standard diet.

### 2.5. Differential Protein Carbonylation Levels Induced by ω-3 Fish Oil

#### 2.5.1. Total Protein Carbonylation

**Figure 1:**
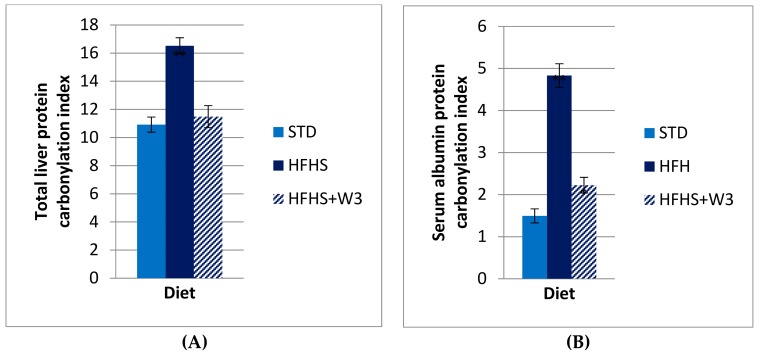
*Protein carbonylation index*: (**A**) total protein carbonylation index in liver from STD, HFHS and HFHS + ω3-fed rats; and (**B**) plasma albumin carbonylation index from STD, HFHS and HFHS + ω3-fed rats. Data are presented as mean ± standard deviation. * Pb.05, ** Pb.01.

**Figure 2:**
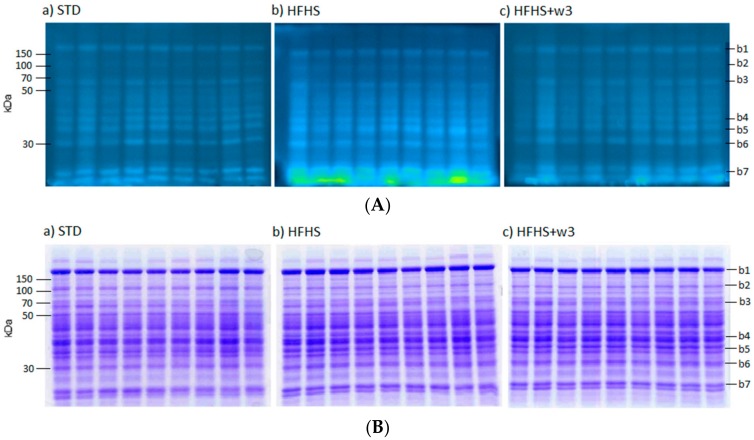
(**A**) *1-D FTSC-stained gel of liver proteins*. Dietary groups: (a) STD; (b) HFHS; and (c) HFHS + ω3. Marked bands (b1–b7) showed differential protein carbonylation index. (**B**) *1-D Coomassie-stained gel of liver proteins.* Dietary groups: (a) STD; (b) HFHS; and (c) HFHS + ω3. Marked bands (b1–b7) showed differential protein carbonylation index.

The analysis of 1-D gels of liver proteins showed significant differences in the levels of global protein carbonylation of STD vs HFHS diet. As evidenced in Figure 1, long-term HFHS consumption triggered a significant increase of global protein carbonylation level in liver (Pb.001). Protein bands were more carbonylated in HFHS than STD rats for most oxidized ones as is exemplified for bands named b1–b7 (Figure 2A,B). Interestingly, such carbonylation was considerably diminished by the supplementation of marine lipids, managing to reach values similar to those of the group fed STD. This result was in agreement with serum albumin carbonylation (Figure 1A,B).

#### 2.5.2. Specific Protein Carbonylation

**Figure 3:**
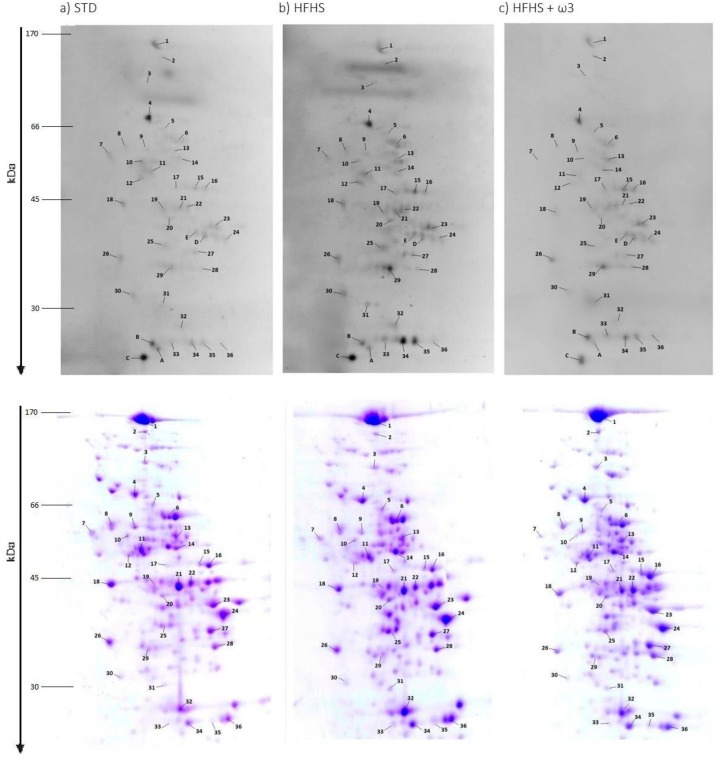
2-D FTSC-stained and Coomassie gels of liver proteins. Dietary groups: (**a**) STD; (**b**) HFHS; and (**c**) HFHS + ω3. Numbered protein spots represent carbonylated proteins confidently identified.

Liver proteins exhibited different susceptibility to the oxidative damage, 2-D gels were performed to deepen the characterization of carbonylated proteins. They allowed isolating and identifying proteins and thus obtaining detailed information regarding the effects of marine lipids on the oxidation levels of every single protein. Despite a large number of visualized protein spots on the Coomassie-stained gels (~200), only a minor portion was distinctively attached to carbonyl-specific FTSC-tag showing a visible carbonylation. These proteins were marked by numbers on the gels (Figure 3a–c). In total, 36 carbonylated spots were visualized, analyzed and identified by MS/MS. 

It is important to highlight that protein vulnerability to oxidation was not directly related to protein concentration. Several protein spots presented at high concentration did not show any detectable carbonylation, whereas other protein spots with strong carbonylation were not even observed in the Coomassie-stained gels. This is the case of proteins named A–E. Protein identifications and the changes in protein carbonylation levels among diets are shown in Table 6.

Protein carbonylation profile was the same in the different dietary groups. However, the normalized carbonylation index (ratio between the carbonylation FTSC signal and the total protein Coomassie intensity) indicated that HFHS consumption increased selectively liver protein carbonylation, showing differences (*p* < 0.05) in 20 spots: Spot 1, Carbamoyl-phosphate synthase (Cps1); Spot 3, Dimethylglycine dehydrogenase (Dmgdh); Spot 4, Serum albumin (Alb); Spot 6, Catalase (Cat); Spot 7, Protein disulfide-isomerase A3 (Pdia3); Spot 8, 60 kDa heat shock protein (Hspd1); Spot 10, Formimidoyltransferase-cyclodeaminase (Ftcd); Spot 11, Aldehyde dehydrogenase (Aldh2); Spot 20, Fumarylacetoacetase (Fah); Spot 21, Argininosuccinate synthase (Ass1); Spot 22, in which two different proteins were mingled, namely Phosphoglycerate kinase (Pgk1) and 3-ketoacyl-CoA thiolase (Acaa2); Spot 24, Fructose-bisphosphate aldolase B (Aldob); Spot 25, which included 3-oxo-5-beta-steroid 4-dehydrogenase (Akr1d1) and Ornithine carbamoyltransferase mitochondrial (Otc); Spot 26, Regucalcin (Rgn); Spot 29, Malate dehydrogenase cytoplasmic (Mdh1); Spot 32, Carbonic anhydrase 3 (Ca3); Spots 33, 34 and 35, Glutathione S-transferase Mu 2 (Gstm2); and Spot 36, 3-hydroxyacyl-CoA dehydrogenase type-2 (Hsd17b10).

Interestingly, the level of carbonylation of each of these individual proteins was modulated by the consumption of marine lipids. Outstandingly, fish oil supplementation generated significant changes in the carbonylation index of several proteins, 27 spots in the HFHS diet. Most of these proteins, which were found more significantly carbonylated after HFHS intake, decreased their carbonylation level when marine lipids were supplemented. Therefore, the carbonylation level of 25 protein spots corresponding to the marine lipids group was significantly lower compared to the control HFHS dietary group. Only two proteins presented a significant higher carbonylation index after marine lipids supplementation: Spot 7 identified as Protein disulfide-isomerase A3 (Pdia3) and Spot 26 (Regucalcin (Rgn)). In contrast to the liver proteins described above, the oxidative levels of other proteins were not affected by the dietary interventions and the marine lipids supplementation either as can be seen in Table 6.

As a notable finding, five spots, tagged in Figure 3 with letters A–E, exhibited high fluorescence intensity in all dietary groups. Nonetheless, it was impossible to calculate the carbonylation index due to their low abundant in the gel spots. It was not practicable to detect them in the Coomassie gels (detection range of Coomassie Blue ~100 ng), and therefore, to elucidate if there was any influence of the dietary framework or the marine lipids on its oxidative level. The carbonylation indexes for these spots should be extremely high considering the high fluorescence signal of these spots (well above the saturation intensity level) and their low protein amount. 

In an attempt to identify these unstained proteins, spots were manually excised directly from the gel under exposition to a UV transilluminator. Spots were digested with trypsin and analyzed with MS/MS. MS/MS analysis revealed the following identifications: Spot A, Peroxiredoxin-6 (Prdx6); Spot B Glutathione S-transferase Mu 1 and 2 (Gstm1 and Gstm2), Triosephosphate isomerase (Tpi1), and Enoyl-CoA hydratase mitochondrial (Echs); and Spot C Protein/nucleic acid deglycase DJ-1 (Park7) and Protein ABHD14B (Abhd14b). Proteins from Spots D and E could not be identified.

### 2.6. Cellular Distribution, Molecular Function and Biological Process of Carbonylated Proteins

**Figure 4:**
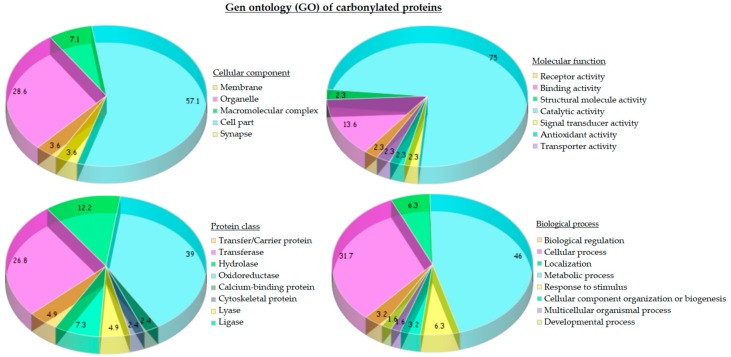
Gene ontology (GO) of carbonylated proteins from liver of both STD and HFHS diets: (**a**) cellular distribution; (**b**) molecular function; (**c**) protein class; and (**d**) biological process of carbonylated proteins.

The statistical analysis of gene ontology (GO) of carbonylated proteins from the liver of both standard and HFHS diets provided a general overview of their cellular distribution, molecular function and biological process (Figure 4). Protein targets of carbonylation were essentially proteins from mitochondria (28.6%) and cytoplasm (57.1%). Considering the molecular function, three-quarters of carbonylated proteins possessed catalytic activity (75.0%) mainly corresponding with oxidoreductase (45%) and transferase (25%) activity, followed, in less proportion, by hydrolase (10%) and ligase activity (7.5%).

Carbonylated proteins with oxidoreductase activity were identified as 6-phosphogluconate dehydrogenase (Pgd), Peroxiredoxin-6 (Prdx6), Dimethylglycine dehydrogenase (Dmgdh), Alcohol dehydrogenase 1 and 2 (Adh1 and Aldh2), Glyceraldehyde-3-phosphate dehydrogenase (Gapdh), Dihydrolipoyl dehydrogenase (Dld), Methylmalonate-semialdehyde dehydrogenase (Aldh6a1), 3-hydroxyacyl-CoA dehydrogenase type-2 (Hsd17b10), 3-oxo-5-beta-steroid 4-dehydrogenase (Akr1d1), Long-chain specific Acyl-CoA dehydrogenase (Acadl), Malate dehydrogenase (Mdh1), Glutamate dehydrogenase 1 (Glud1), Transketolase (Tkt), Catalase (Cat), 3-hydroxyanthranilate 3,4-dioxygenase (Haao), Alpha-aminoadipic semialdehyde dehydrogenase (Aldh7a1), and Homogentisate 1, 2-dioxygenase (Hgd). 

Proteins exhibiting transferase activity were subdivided in turn into those that transfer acyl groups (3-ketoacyl-CoA thiolase (Acaa2) and Hydroxymethylglutaryl-CoA synthase (Hmgcs2)), with kinase activity (Glycerol kinase (Gk) and Phosphoglycerate kinase 1 (Pgk1)), transaminase activity (4-aminobutyrate aminotransferase (Abat)) and transketolase activity (Transketolase (Tkt)). In the group of proteins with hydrolase activity, we found Regucalcin (Rgn), Carbamoyl-phosphate synthase (Cps1), Formimidoyltransferase-cyclodeaminase (Ftcd), and Beta-ureidopropionase (Upb1), and with ligase activity Argininosuccinate synthase (Ass1) and Pyruvate carboxylase (Pc). Regarding biological process, almost half of carbonylated proteins (46.0%) were involved in metabolic process; more concretely, 36.4% proteins were involved in cellular amino acid metabolism, 21.2% in lipid metabolism and 18.2% in carbohydrate metabolism.

### 2.7. Network Analysis

**Figure 5:**
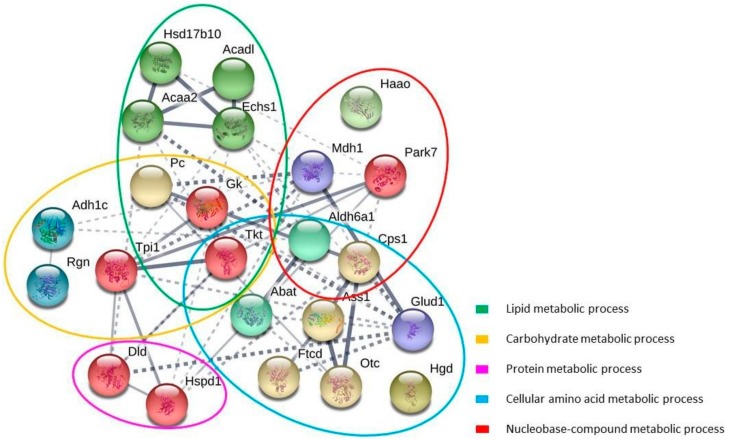
Network analysis of carbonylated proteins from the liver of both STD and HFHS diets. Graphic representation of the network of connections of all proteins that appear carbonylated and were identified in liver.

STRING database analysis showed a network composed by 23 nodes (proteins) and 52 interactions (Figure 5). The STRING associations illustrated the functional interactions between the carbonylated proteins and their potential physiological significance. The topological analysis of the network revealed the existence of several subnetworks, in concordance with the main biological functions: lipid, carbohydrate, protein, cellular amino acid and nucleobase-compound metabolic process. Inside the subnetworks of lipid metabolism and carbohydrate metabolism, the proteins of interest showed highly connected interactions and three of them are common to both groups: Pyruvate carboxylase (Pc), Glycerol kinase (Gk), Transketolase (Tkt). Methylmalonate-semialdehyde dehydrogenase (Aldh6a1) and Carbamoyl phosphate synthase (Cps) were the common proteins in the case of the subnetworks of cellular amino acid metabolism and nucleobase-compound metabolism

It is worth noting that the protein Transketolase (Tkt) (an enzyme of the pentose phosphate pathway) was closely connected with most proteins of interest, playing a central role in the Network and stating a close relationship between the different subnetworks. 

In both lipid metabolism and cellular amino acid metabolism subnetworks, some proteins that constituted a central interplay with high interconnections could be seen. In the first case, those proteins were 3-hydroxyacyl-CoA dehydrogenase (Hsd17b10), Long-chain specific acyl-CoA dehydrogenase (Acadl), 3-ketoacyl-CoA thiolase (Acaa2) and Enoyl-CoA hydratase (Echs1) and all of them belong to the mitochondrial β-oxidation, which plays a major role in energy production during periods of physiologic stress. In the second subgroup, the proteins were the first three enzymes participating in the urea cycle, i.e., Carbamoyl phosphate synthase (Cps), Ornithine carbamoyltransferase (Otc) and Argininosuccinate synthase (Ass1).

## 3. Discussion

The research carried out in this work tried to explore the way that marine lipids supplementation can counteract the deleterious effects of the consumption of high-caloric diets, mainly from the perspective of the oxidative stress and proteins. Protein carbonylation, one of the most harmful irreversible oxidative protein modifications, is considered as a major hallmark of oxidative stress-related disorders. Therefore, this study of oxidative stress and diet was addressed by the use of this stable post-translational modification of proteins. Liver, as a critical organ in the metabolism of fats, was selected as the target for studying the differential modulation of its redox proteome through the diet fat composition. 

As the first outcome, the consumption of the HFHS diet led to metabolic alterations in rats like high adiposity index, hyperinsulinemia and significant deterioration of the antioxidant status because of both higher levels of oxidants and lower antioxidant activities. The higher level of plasma insulin required for maintaining the normal glucose levels actually is the earliest sign of the onset of type-2 diabetes in HFHS-rats [14] and it is in agreement with our previous results [9,12,15]. The higher adiposity index of these rats was mainly due to a disproportional increase in the visceral adipose depot (perigonadal), which is associated with cardiovascular diseases and non-alcoholic fatty liver disease. The hyperinsulinemia measured in HFHS-fed rats could also favor this adipose tissue hypertrophy, since insulin inhibits lipolysis and promotes lipogenesis in adipocytes [16]. However, none parameters indicated the development of fatty liver induced by the long-term consumption of the HFHS diet even if the development of hepatic steatosis has been numerously reported in rodents fed high-fat diets [17]. In this early step of the metabolic alteration development, the visceral adipose tissue in HFHS-fed rats may still preserve the ability to the absorption of circulating free fatty acids and prevent their accumulation in the liver. 

Additionally, lower activity of ALT and AST was found after the long-term HFHS intake. Since ALT and AST are expressed in other organs but liver, these low blood levels below the normal STD value could be related with some other alterations present in HFHS rats. Low AST and especially low blood ALT levels are associated with sarcopenia, frailty and increased long-term mortality in patients suffering from coronary heart disease [18]. The inclusion of fish oil in the HFHS counteracted in part the reduced activity of AST, but more investigation is needed to fully understand the modulation of AST and ALT activities by fish oils and the deregulation found in our study. 

Data corresponding to lipid peroxidation and total protein carbonylation corroborated that the metabolic alterations caused by the long-term consumption of the HFHS diet, coursed with an increment of oxidative stress, which affected liver lipids and proteins. More specifically, many of the 20 proteins whose carbonylation level was altered by HFHS possessed oxidoreductase activity and were involved in the response to oxidative stress and the antioxidant defense. Oxidative alterations in these proteins pointed out the rupture of the liver redox homeostasis induced by HFHS diet, which was previously reported in both a model of metabolic alteration induced by HFHS diet [9] and in a genetic model of metabolic syndrome [19]. According to these results, it was found significant impairment in the antioxidant liver status of these HFHS-fed rats. 

The protective effect of fish oil detected on the liver was mainly focused on reducing the oxidative imbalance while improving the pro-inflammatory phenotype promoted by the consumption of the HFHS diet. 

First, the supplementation with marine lipids increased the accumulation of ω-3 EPA, DPA and DHA in the liver while decreasing the proportion of ARA. As a final consequence, the ω-6/ω-3 ratio was lower in the HFHS diet supplemented with marine ω-3 PUFA, being this ratio considered as an excellent clinical marker for cellular inflammation [20]. Marine lipids supplementation also tended to reduce the amount of other inflammatory markers, such as 18:1ω9 and 16:1ω7. Increased amounts of oleic acid in tissues have been linked to obesity [13], hypertriacylglycerolemia and the risk of developing insulin resistance [21], conditions which are usually accompanied by low-grade chronic inflammation and increased oxidative stress [22]. This finding is in agreement with previous results found in other rat strains fed mixtures of fish oils and grape polyphenols [12]. 

Regarding oxidative stress, marine lipids markedly reduced the total level of protein carbonyls. Moreover, the detailed analysis of individual protein carbonylation levels highlighted the ability of ω-3 PUFA to counteract the effect of HFHS diet. 

An important set of these proteins were involved in the redox balance. Accordingly, most of the antioxidant enzymes involved in the in vivo defense system were more active in rats fed HFHS diet supplemented with marine fatty acids. Considering that the level of polyunsaturation per gram of liver was significantly higher after marine lipids supplementation due to the increase in the concentration of ω-3 PUFA, there was an increment of the oxidizable substrate. The well-known overproduction of ROS associated to high-caloric diets and the higher number of unsaturations coming from the ω-3 PUFA supplement could make liver lipids more susceptible to lipid peroxidation, which seemed to result on an up-regulation of the antioxidant system. 

Among the proteins directly related to the antioxidant system, such as catalase, it is especially noteworthy the decrease in the carbonylation of Aldh2, a key enzyme in the protection against cardiovascular risk because it eliminates free aldehydes such as 4-HNE in the mitochondria [23]. Previous studies have found increased carbonylation level of this protein associated to metabolic alterations related to diet [9,19]. The present work demonstrated that marine lipids specifically reduced the carbonylation level of this protein, which it is postulated as an important target in the cardiovascular protection exerted by marine lipids.

Less oxidative damage of the albumin and Akr1d1 also indicated an improvement of the antioxidant status by marine lipids and a possible protective effect in the face of the development of long-term pathologies linked to oxidative stress. On one hand, albumin is the main antioxidant in plasma [24]. On the other hand, Akr1d1, a NADPH-dependent enzyme [25], participates in the reductive regeneration of ascorbic acid in the liver, especially under oxidative stress [26]. Down-regulation in the Akr expression in the liver of diabetic rats caused a reduction in ascorbic acid regeneration and the subsequent increment of oxidative stress [27]. In the same way, oxidized Akr could provoke a reduction of the ascorbic acid level and antioxidant capacity. Therefore, the lower carbonylation level of Ark given by ω-3 PUFA might provide a correct regulation of ascorbic acid regeneration that directly influences the antioxidant balance [28]. 

Besides the proteins that directly control the redox homeostasis, marine lipids were able to modulate several novel functional proteins involved in the regulation of lipid, carbohydrate, one-carbon, citric acid cycle and protein metabolism, suggesting an integrated regulation of metabolic pathways. Dehydrogenase Acadl, essential for fatty acid oxidation, metabolizing fats and converting them to energy [29] and Acaa2, which can metabolize long-chain fatty acids found in the marine lipid supplement, were two of the proteins from lipid metabolism modulated by fish oil. 

EPA and DHA also displayed an important effect on proteins of nitrogen metabolism. Among them, Cps1, which is a known target of carbonylation in the liver [30] and Ass1, which acts in the ammonia detoxification and whose expression is submitted to both hormonal and nutritional regulation. The carbonylation of liver Ass1 observed under HFHS diets could affect the optimal removal of toxic ammonia, and finally, would lead to metabolic disturbances as hyperammonemia [31]. Additionally, the protective effect of ω-3 PUFA on Otc can provide a right nitrogen metabolism. Ahmed et al. [32] has recently described a significantly increased in both expression and activity of the ornithine aminotransferase in ω-3 PUFA enriched diets. 

More carbonylation of Regucalcin was found in ω-3 PUFA supplemented HFHS group. This protein plays a role in the maintenance of cytosolic Ca2+ homeostasis in liver cells. Ahmed et al. [32] previously reported a lower expression of Regucalcin in mice fed a high-ω-3 PUFA diet compared to mice fed a low-ω-3 PUFA one. Higher expression of Regucalcin has been liked to adipogenesis in adipocytes [33], and also alterations in lipid and glucose metabolism in vivo, which are predisposing factors to obesity and diabetes. Therefore, the higher liver carbonylation of Regucalcin found in rats fed marine lipids would lead to a decrease in its activity, contributing to the overall beneficial effect found in this work.

As regards carbohydrate metabolism, the lower carbonylation of Aldob is remarkable. Aldob is a critical regulatory enzyme in both the glycolytic and gluconeogenesis pathways [34]. This enzyme was also regulated in the liver of healthy rats by marine ω-3 PUFAs, demonstrating its high vulnerability to respond to diet ω-3 marine lipids [35]. Supplementation with ω-3 PUFA altered proteins involved in citric acid cycle as well. In fact, rats fed EPA and DHA showed lower carbonylation of Mdh1, which catalyzes the oxidation of malate to oxaloacetate to release stored energy derived from carbohydrates, fats, and proteins, so its activity is clearly linked to the diet

Finally, it is important to emphasize the proteins whose carbonylation indexes were impossible to calculate because of the low abundance. Interestingly, all of them perform an essential role in cell redox homeostasis and lipid and glucose metabolism. The µclass of enzymes glutathione S-transferase functions in the detoxification of electrophilic compounds and products of oxidative stress by conjugation with glutathione. Peroxiredoxin-6, a thiol-specific peroxidase, also plays a role in cell protection against oxidative stress by detoxifying peroxides and participates in phospholipid homeostasis. In recent years, α/β-hydrolase domain (ABHD) proteins have emerged as novel potential regulators of lipid metabolism and signal transduction pathways [36]. Enoyl-CoA hydratase acts in the second step of the mitochondrial fatty acid β-oxidation pathway, catalyzing the hydration of 2-trans-enoyl-coenzyme A (CoA) intermediates to L-3-hydroxyacyl-CoAs; while Triosephosphate isomerase 1 catalyzes the isomerization of Glyceraldehyde 3-phosphate (G3P) and dihydroxy-acetone phosphate (DHAP) in glycolysis and gluconeogenesis. The last protein identified was Protein/nucleic acid deglycase DJ-1, which is a protein deglycase that acts on early glycation intermediates preventing the formation of AGEs that cause irreversible damage [37]. It plays an important role in cell protection against oxidative stress and cell death acting as an oxidative stress sensor and a redox-sensitive chaperone and protease [38]. It eliminates hydrogen peroxide and protects cells against hydrogen peroxide inducing-cell death [39]. 

## 4. Materials and Methods

### 4.1. Materials and Reagents

Fish oil diet supplement was obtained by mixing commercial fish oils AFAMPES 121 EPA (AFAMSA, Vigo, Spain) and EnerZona Omega 3 RX (Milan, Italy) with 1:1 EPA:DHA ratio and EPA+DHA 50% of TFA. Fatty acid composition was assayed by gas-liquid chromatography showing a soybean oil, obtained from unrefined organic soy oil was from Clearspring Ltd. (London, UK). The supplemented doses of fish oil agreed with the European Union’s recommendation on ω-3 PUFA

Protease inhibitor ProteoBlock was obtained from Thermo Fisher Scientific Inc. (Rockford, IL, USA). Ketamine-HCl was purchased from Merial Laboratorios S.A. (Barcelona, Spain). Protein quantifications were made using Bio-Rad protein assay and bicinchoninic acid (BCA) assay from Sigma (St. Louis, MO, USA). Fluorescein-5-thiosemicarbazide (FTSC) labeling for fluorescent imaging was purchased from Invitrogen (Carlsbad, CA). Trypsin sequencing-grade from Promega (Madison, WI, USA) was used for protein digestion. Acrylamide and *bis*-*N*,*N*-methylene-*bis*-acrylamide were obtained from Bio-Rad Laboratories (Hercules, CA, USA). For 2-D electrophoresis were used Immobiline DryStrip gels (IPG strips) for isoelectric focusing (IEF) of pH range 3–10 and lengths 11 and 18 cm, IPG buffer, pharmalyte 3–10, bromophenol blue and TEMED, all of them purchased from GE Healthcare Bio-Sciences AB (Uppsala, Sweden). Internal standard of nonadecanoic acid (19:0) was acquired from Larodan Fine Chemicals (Malmö, Sweden). Phenylmethylsulfonyl fluoride (PMSF), dithiothreitol (DTT), iodoacetamide (IA), ethylenediaminetetraacetic acid (EDTA), trichloroacetic acid (TCA), Tris Hydrochloride (Tris–HCl) and CHAPS detergent were obtained from Sigma (St. Louis, MO, USA). 

### 4.2. Animals 

Twenty-seven male 3–4-week-old Sprague-Dawley rats weighing about 50 g (Harlan Laboratories Ltd., Derby, UK) were kept in an insulated room with a constantly regulated temperature (22 ± 2 °C) and controlled humidity (50 ± 10%) in a 12 h artificial light cycle. The animals were randomly assigned to three diet groups: a control standard diet group (STD) (*n* = 9) fed a standard diet (Teklad Global 14% Protein Rodent Maintenance Diet, Harlan Laboratories, Derby, UK); a high fat high sucrose diet group (HFHS) (*n* = 9) fed a high caloric diet (TD.08811 45% kcal Fat Diet, Harlan Laboratories, Derby, UK) and a HFHS group supplemented with marine ω-3 PUFA (±0.8 mL/Kg body weight EPA/DHA 1:1 per week). Fatty acid composition of the diets is shown in Tables S1 and S2 and it is the same used by Dasilva et al. [12]. 

Rats had ad libitum access to water and food. Food intake and water consumption were registered daily throughout the study. Rats were sacrificed by exsanguination after being anesthetized intraperitoneally with ketamine and xylacine (80 mg/kg and 10 mg/kg body weight, respectively).

All the procedures agreed with the European Union guidelines for the care and management of laboratory animals. The pertinent permission for this study was obtained from CSIC (Spanish Research Council) Subcommittee of Bioethical Issues 

### 4.3. Fatty Acid Analysis of Diets

The fatty acid composition of diets was determined following the method of Lepage and Roy after the extraction of lipids (Bligh and Dyer, [40]). Briefly, 0.6 mg of lipid were methylated and analyzed by gas chromatography (GC/FID; Clarus 500; PerkinElmer, Waltham, MA, USA) Fatty acid nonadecanoic acid (19:0) was used as an internal standard. The identification of the different fatty acids was accomplished by comparison of retention times with a mixture of standards. Results were expressed as a percentage of total fatty acids (Table S2).

### 4.4. Plasma and Tissue Sample Collection

Blood from each animal was centrifuged at 850× *g* (15 min at 4 °C) to remove erythrocytes. Then, plasma samples were immediately stored with 5 mM PMSF (protease inhibitor) at −80 °C. Liver was excised, washed with 0.9% NaCl solution, weighed and immediately frozen in liquid nitrogen upon sacrifice. The hepatosomatic index [(liver weight × 100)/body weight] was determined. Samples were stored at −80 °C until analysis.

### 4.5. Plasmatic Biochemical Measurements

Triglycerides, total cholesterol, LDL- and HDL-cholesterol were measured in plasma by enzymatic/colorimetric methods (SpinReact Kits, Girona, Spain) as described by Bucolo et al. The intensity of the color formed was proportional to the compound concentration [41]. 

Glycemic status was analyzed measuring fasting blood glucose and plasma insulin levels. Blood glucose concentration was measured by the enzyme electrode method, using an Ascensia ELITE XL blood glucose meter (Bayer Consumer Care, Basel, Switzerland); plasma insulin levels were measured using Milliplex xMAP multiplex technology on a Luminex xMAP instrument (Millipore, Austin, TX, USA).

Serum ALT and AST levels were measured on fasting morning plasma samples using the kinetic UV method (Liquid-Stat Reagent Kit Beckman Coulter, Nyon, Switzerland)

### 4.6. Total Fatty Acids Analysis in Liver

Lipid extraction was performed according a modification of Bligh and Dyer protocol with dichloromethane:methanol:water (2:2:1, *v*/*v*) as extraction solvent. Gravimetric quantification was carried out so that 0.6 mg of organic phase was transesterified and TFA were analyzed by gas chromatography (GC/FID, Clarus 500, PerkinElmer).

### 4.7. Fatty Acid Desaturase (FAD) Indexes

FAD indexes were calculated from liver TFA. 

Δ9 Stearoyl-CoA Desaturases SCD-16 and SCD-18 regulate the desaturation of SFA to MUFA, specifically from palmitic acid (16:0) into palmitoleic acid (16:1ω7) and stearic acid (18:0) into oleic acid (18:1 ω-9), respectively. 

Δ4, Δ5, and Δ6 Desaturases (Δ4D, Δ5D, and Δ6D) are involved in the metabolism of linoleic (LA) and α-linolenic (ALA) acids to PUFAs ARA, EPA and DHA. The pathway from ALA (18:3ω3) into EPA (20:5ω3) is mediated by the action of both Δ5D and Δ6D. 

The activity of desaturase enzymes was estimated from the concentration ratio of the enzyme product to its substrate as follows: SCD-16 = [16:1ω − 7/16:0], SCD-18 = [18:1ω − 9/18:0], Δ4D = [22:6ω3/22:5ω3], Δ5D = [20:4ω6/20:3ω6], Δ6D = [20:3ω6/18:2ω6] and Δ5/6D = [20:5ω3/18:3ω3].

### 4.8. Determination of Lipid Peroxidation Levels and Antioxidant Systems in Blood and Liver Samples

Lipid peroxidation levels in liver were measured through conjugated dienes hydroperoxides (intermediate lipid oxidation product) following the American Oil Chemists Society method. Liver lipids were extracted and quantified. Then, conjugated dienes were measured using a spectrophotometer set at 234 nm. 

Total superoxide dismutase (SOD), Catalase (CAT), Glutathione peroxidase (GPx) and glutathione reductase (GR) activities were determined according to previously described spectrophotometric methods [42,43,44].

Plasma antioxidant capacity was measured as the oxygen radical absorbance capacity (ORAC). Oxidized and reduced glutathione balance (GSSG/GSH) was measured by the Hissin and Hilf fluorometric method at wavelengths of 350 nm (excitation) and 420 nm (emission) [45].

### 4.9. Protein Extraction

For the extraction of proteins, 300 mg of liver were homogenized by sonication for 1 min under 0.6 s cycle and 100% of amplitude (Labsonic sonifier from Sartorius-Germany). The homogenate was centrifuged at 100,000× *g* (60 min at 4 °C) to recover proteins that remain in the supernatant solution. The bicinchoninic acid assay (BCA) was used for quantitation of total protein.

### 4.10. FTSC Labeling of Protein Carbonyls

Protein carbonyl groups generated in vivo were derivatized by FTSC and detected through the measurement of carbonyl-derivative fluorescence to evaluate the influence of the different diets on rat protein oxidation. Proteins extracted as described above were incubated with 1 mM FTSC in dark (37 °C, 2.5 h). Afterwards, proteins were precipitated with an equal volume of 20% TCA (*v*/*v*), centrifuged at 16,000× *g* (20 °C, 10 min) and redissolved in urea buffer (7 M urea, 2 M thiourea, 2% Chaps, 0.5% Pharmalyte 3–10, 0.5% IPG 3–10 buffer, and 0.4% DTT). Quantification of protein concentration was measured by Bradford assay.

### 4.11. Gel Electrophoresis SDS–PAGE (1-D and 2-D)

#### Total and Specific Protein Carbonylation

To evaluate the global protein carbonyl levels in liver, 30 μg of each FTSC-labeled sample were separated in a monodimensional (1-D) 10% SDS-polyacrylamide gel electrophoresis (PAGE) and run in a Mini-PROTEAN 3 cell (Bio-Rad, Hercules, CA).

To study the protein carbonyl levels of individual proteins, FTSC-labeled proteins were resolved in bidimensional (2-D) electrophoresis gel. Briefly, 11-cm or 18-cm IPG dry strips with pH range 3–10 (Immobiline DryStrip gels, GE Healthcare Science, Uppsala, Sweden) were loaded with 400 μg of protein sample. Isoelectric focusing (IEF) was performed at 20 °C with an Ettan IPGphor II isolectric focusing system (GE Healthcare Science) using a programmed voltage gradient.

Prior to run the second dimension, strips were incubated for 15 min in equilibration buffer (6 M urea, 2% SDS, 50 mM Tris–HCl, pH 8.8, 30% glycerol) first with 0.075% DTT and then with 4.5% iodoacetamide. The equilibrated IPG strips were inserted onto 2-D laboratory-made 10% SDS-PAGE gels. The electrophoresis was run at 10 mA/gel for 1 h and 20 mA/gel for approximately 12 h at 15 °C using an Ettan Daltsix electrophoresis system (GE Healthcare Science).

To visualize FTSC-tagged proteins, 1-D or 2-D gels were exposed to a UV transilluminator UVP BioDoc-It2 Gel Imaging System (Analytik Jena AG, Upland, CA, USA) equipped with a 520-nm band-pass filter (520DF30 62 mm). Finally, gels were stained overnight with Coomassie dye PhastGel Blue R-350 (GE Healthcare Science) and washed to remove background staining to visualize total protein level in each sample.

### 4.12. Image Analysis

The analysis of total protein carbonylation (1-D) was performed using LabImage 1D (Kapelan Bio-Imaging Solutions, Halle, Germany). Specific protein carbonylation (2-D) was quantified with PDQuest software version 7.4 (Bio-Rad, Hercules, CA, USA). The intensity of the spots was expressed as parts per million of the total integrated optical density of the gel

### 4.13. In-Gel Digestion and Peptide Extraction and Protein Identification by nanoLC–ESI–IT–MS/MS

Protein spots were manually excised from 2-D-SDS PAGE and consecutively washed with water and dehydrated in 100% acetonitrile. Each spot was enzymatically digested overnight at 37 °C with a 0.5 μM solution of sequencing-grade trypsin (Promega, Madison, WI, USA) in 50 mM NH_4_HCO_3_ buffer. 

Afterwards, the tryptic peptides were desalted by using C18 ZipTips (Millipore) and reconstituted up in 1% formic acid. 

The chromatographic separation was carried out on a Dionex UltiMate 3000 Series (Thermo Fisher, Rockford, IL, USA) on a C18 column (Acclaim PepMap RSLC C18, 2 μm, 100 Å, 75 μm i.d. × 15 cm) with a trap-column (μ-Precolumn holder, 5 mm, with 30 μm i.d.) (Thermo Scientific, San Jose, CA, USA) using a binary eluent system of water 0.1% (*v*/*v*) of formic acid (phase A) and acetonitrile 0.1% (*v*/*v*) of formic acid (phase B). uHPLC system was coupled to a dual-pressure linear ion trap mass spectrometer LTQ Velos Pro with electrospray ionization (ESI) (Thermo Fisher, Rockford, IL, USA). Peptides were separated with a 90-min linear gradient from 5% to 40% B at a flow rate of 0.300 μL/min. 

Survey scans were acquired in the mass range of *m*/*z* 400 to 1600 Da followed by MS/MS scans of the 6 most intense peaks. These 6 most intense peaks with ≥2 charge state were selected in the ion trap for fragmentation by collision-induced dissociation with 35% normalized collision energy and an isolation width of 2 Da. Dynamic exclusion was enable for 30 s after the second fragmentation event. Instrument control and data acquisition was controlled by Xcalibur 2.0 and Tune 2.2 software (Thermo Fisher Scientific, Inc.).

Protein identification was performed by homology of experimental MS/MS peak lists with theoretical MS/MS spectra contained in the *Rattus norvegicus* UniProtKB/Swiss-Prot database (29,969 sequences) released in April 2018 by using PEAKS DB protein identification (Bioinformatics Solutions Inc., Waterloo, ON, Canada). 

Carbamidomethylation of cysteine and methionine oxidation were set as variable modifications. Trypsin was used as proteolytic enzyme and only 2 missed cleavages sites were allowed per peptide. For MS/MS events precursor and fragment ions tolerance were set to ±1.5 Da and ±0.8 Da, respectively. For decoy database search, False Discovery Rate (FDR) targets were set at <1%.

### 4.14. Statistical Analysis/Calculation of Carbonylation Indexes

To analyze the influence of diet on protein carbonylation level, “protein carbonylation index” was calculated. 

This normalized parameter compares:

(1) $$\;\mathrm{protein}\;\mathrm{carbonylation}\;\mathrm{index}=\frac{\mathrm{spot},\;\mathrm{band}\;\mathrm{or}\;\mathrm{lane}\;\mathrm{fluorescence}\;\mathrm{intensity}\;\mathrm{in}\;\mathrm{the}\;\mathrm{FTSC}\;\mathrm{stained}\;\mathrm{gel}\;}{\mathrm{spot},\;\mathrm{band}\;\mathrm{or}\;\mathrm{lane}\;\mathrm{intensity}\;\mathrm{in}\;\mathrm{the}\;\mathrm{Coomassie}\;\mathrm{stained}\;\mathrm{gel}}\;$$

Individual and global oxidation protein levels were reported as mean and standard deviation (s.d.). 

Statistical analyses were performed by analysis of variance (ANOVA) with IBM SPSS Stadistics version 24 software (SPSS, Chicago, IL, USA). Normal distribution and homogeneity of variance were evaluated. Nonparametric Kruskal–Wallis analyses were applied when data distribution did not fit a Gaussian model or heterogeneity was found in variances. The means were further compared by the post-hoc test Fisher least square difference (Fisher LSD). The level of significant difference was set at *p* < 0.05.

### 4.15. Functional Pathways, Gene Ontology (GO) Analysis and Interaction Network Analysis

Protein IDs list (Gene name) was submitted to PANTHER version 13.1 (Protein ANalysis THrough Evolutionary Relationships) (http://www.pantherdb.org/) (University of Southern California, CA, USA), for the classification based on two main types of annotations: protein class and biological process. The whole *Rattus norvegicus* genome was selected as a reference set.

### 4.16. Network Analysis

Network analysis of the identified carbonylated proteins was performed submitting the *Rattus norvegicus* gene IDs to the STRING software version 10.5 (Search Tool for the Retrieval of Interacting Genes) (http://stringdb.org/) (Academic Consortium), a database of known and predicted protein interactions.

Proteins were represented with nodes. Continuous lines represent physical interactions of the proteins and interrupted lines represent functional interactions.

All edges were supported by at least a reference from the literature or from canonical information stored in the STRING dataset. A confidence score was fixed to 0.4 (medium level). Cluster networks were created using the MCL algorithm and a value of 4 was selected for all the analyses.

## 5. Conclusions

The present study focused on the identification of a pattern for molecular alterations presented under HFHS diet framework and early stages of oxidative damage. It provided evidence of the effect of fish oils to reduce the liver protein carbonylation and pro-inflammatory phenotype, as well as the enhancement of the endogenous antioxidant system. Marine ω-3 PUFA consumption led to specific protection of liver proteins as well. This different susceptibility of proteins to be oxidized together with the fact that carbonylation did not depend on the concentration of the protein, support the lack of randomness of protein oxidation being more than a mere reflection of ROS levels. In fact, there were highly abundant proteins without any carbonylation, or very negligibly, coexisting with low concentrated but extremely carbonylated proteins. Another fact that emphasized the specificity of the carbonylation resides on the different behavior found over some liver proteins, which were found with lower levels of carbonylation after supplementation with EPA and DHA, demonstrated a selective capacity of response of the hepatic proteome to the protection of carbonylation exerted by marine lipids. The identification of the target carbonylation proteins on which marine ω-3 PUFA exerted a significant effect, allowed the identification of pathways potentially modulated by these acids. Thus, fish oils led to a significant protection of enzymes with hepatic oxidoreductase capacity, enzymes related to transport, absorption and oxidation of fatty acids and amino acid catabolism. In conclusion, the fat quality in terms of the intake of marine fatty acids plays a critical role on liver protein regulation of oxidative stress linked to the consumption of high caloric diets.

## Figures and Tables

**Table 1:** Morphological measurements and biochemical parameters from Sprague-Dawley rats with different diets. HDL, high-density lipoprotein cholesterol; LDL, low-density lipoprotein cholesterol; AST, aspartate aminotransferase; ALT, alanine aminotransferase.

Parameter	STD		HFHS		HFHS + ω3	
	Mean	s.d. ^c^	Mean	s.d. ^c^	Mean	s.d. ^c^
Body weight after 24 weeks	539.6	49.4	568.3 *	24.3	579.9 *	35.7
Length	25.7	0.4	26.5	0.6	26.4	0.4
Perigonadal Adipose Tissue	9.0	3.2	13.1	3.9	13.3	4.4
Adiposity index (%) ^a^	1.7		2.3 **		2.3 **	
Hepatosomatic index (%) ^b^	0.50		0.44		0.43	
Liver weight	2.7		2.5		2.5	
Liver fat content	7.01	0.61	6.64	0.79	6.55	0.70
Triglycerides (mmol/L)	0.69	0.20	0.52	0.26	0.59	0.23
Cholesterol (mmol/L)	3.61	0.38	2.90	0.50	2.55	0.42
HDL (mmol/L)	1.15	0.12	0.94	0.16	0.85	0.12
LDL (mmol/L)	0.43	0.11	0.39	0.09	0.32	0.07
Plasmatic Insulin ng/mL	0.95	0.6	2.0 *	0.25	2.1 *	0.40
Plasmatic Glucose mg/mL	65	2	66	1.5	64	2
AST (U/L)	70.19	21.91	57.12 *	10.74	53.58 *	10.15
ALT (U/L)	27.09	8.24	19.03 *	3.88	22.84	4.18
AST/ALT	2.64	0.52	3.14	0.92	2.37	0.29
ALT/AST	0.39	0.08	0.35	0.12	0.43	0.05

^a^ Adiposity index: (total abdominal fat × 100)/body weight; ^b^ Hepatosomatic index: (liver weight × 100)/body weight; ^c^ s.d., standard deviation; * Pb.05 vs. standard (STD) control group; ** Pb.01 vs. STD control group.

**Table 2:** Liver Antioxidant System and Redox Status. SOD, superoxide dismutase; CAT, catalase; GR, glutathione reductase; GPx, glutathione peroxidase; GSH, reduced glutathione; GSSG, oxidized glutathione.

Parameter	STD		HFHS		HFHS + ω3	
	Mean	s.d. ^a^	Mean	s.d. ^a^	Mean	s.d. ^a^
SOD (U/g Tissue)	813.11	154.56	579.17 *	58.90	742.72	61.18
CAT (mmol/g Tissue)	16.83	2.61	15.46	0.47	16.22	0.86
GR (U/g Tissue)	157.23	27.43	109.25 *	7.90	134.47	11.32
GPX (U/g Tissue)	848.56	207.92	680.24 *	34.88	766.28	53.36
GSH (µmol/g Tissue)	1.38	0.72	0.73 *	0.16	1.00	0.22
GSSG (µmol/g Tissue)	2.72	0.42	2.36	0.15	1.95	0.15
GSSG/GSH	2.45	1.27	4.51 *	0.94	2.87	0.76

^a^ s.d. standard deviation; * Pb.05 vs. STD control group.

**Table 3:** Fatty acid composition livers expressed in percentage of total fatty acids.

% FA	STD		HFHS		HFHS + ω3	
	Mean	s.d. ^a^	Mean	s.d. ^a^	Mean	s.d. ^a^
14:0	0.39	0.05	1.12 *	0.15	1.06 *	0.12
15:0	0.20	0.01	0.47 *	0.02	0.44 *	0.03
16:0	20.31	0.38	22.07	0.45	21.88	0.43
16:1ω7	1.36	0.20	2.26 *	0.45	1.92 *	0.29
17:0	0.50	0.04	0.58	0.01	0.57	0.01
18:0	9.81	0.53	11.21	0.79	11.70	0.79
18:1ω9	8.24	0.76	19.56 *	1.21	17.66 *	1.10
18:1ω7	3.71	0.12	3.60	0.31	2.76	0.14
18:2ω6	25.93	0.83	10.68 *	0.39	10.82 *	0.28
18:3ω6	0.54	0.03	0.24 *	0.02	0.18 *	0.00
20:0	0.00	0.00	0.00	0.00	0.00	0.00
18:3ω3	0.67	0.05	0.35 *	0.04	0.34 *	0.04
20:1ω9	0.15	0.01	0.50 *	0.06	0.48 *	0.06
18:4ω3	0.00	0.00	0.00	0.00	0.00	0.00
20:2ω6	0.33	0.03	0.10 *	0.01	0.11 *	0.01
20:3ω6	0.34	0.01	0.68 *	0.08	0.86 *	0.05
20:4ω6	20.01	1.10	17.01 *	1.18	15.22 *	0.96
20:5ω3	0.32	0.03	0.37	0.04	1.11 *	0.13
22:4ω6	0.71	0.06	0.29 *	0.03	0.21 *	0.01
22:5ω6	0.44	0.02	0.28 *	0.04	0.16 *	0.01
22:5ω3	1.34	0.06	1.00	0.10	1.41	0.09
22:6ω3	4.69	0.27	7.64	0.65	11.10 *	0.63
SAT	31.22	0.26	35.44	0.22	35.65	0.32
MUFA	13.46	1.02	25.93 *	1.99	22.83 *	1.54
PUFA	55.32	0.96	38.63 *	1.78	41.52 *	1.28
ω-3	7.02	0.25	9.35	0.62	13.96 *	0.57
ω-6	48.30	0.81	29.28 *	1.19	27.56 *	0.84
EPA/DHA	0.068		0.048		0.100 *	
% EPA PUFAs	0.18		0.14		0.46 *	
% DHA PUFAs	2.59		2.95		4.61 *	

^a^ s.d., standard deviation; * Pb.05 vs. STD control group.

**Table 4:** Fatty acid desaturases (FADs) indexes FAD indexes were determined as the ratio between product and precursor.

Desaturases	STD		HFHS		HFHS + ω3	
Ratios	Mean	s.d. ^a^	Mean	s.d. ^a^	Mean	s.d. ^a^
SCD-16 [16:1ω7/16:0]	0.07	0.01	0.10 *	0.02	0.09 *	0.01
SCD-18 [18:1ω9/18:0]	0.87	0.12	1.82 *	0.28	1.57 *	0.21
Δ4D = [22:6ω3/22:5ω3]	3.53	0.21	7.94 *	1.02	8.07 *	0.82
Δ5D = [20:4ω6/20:3ω6]	60.22	4.02	26.90 *	4.24	18.00 *	1.45
Δ6D = [20:3ω6/18:2ω6]	0.01	0.00	0.06	0.01	0.07	0.00
Δ6D = [18:3ω6/ 18:2ω6]	0.02	0.00	0.02	0.00	0.02	0.00
Δ5D/Δ6D = [20:5ω3/18:3ω3]	0.47	0.04	1.06 *	0.06	3.37 *	0.48

^a^ s.d. standard deviation; * Pb.05 vs. STD control group.

**Table 5:** Liver conjugated dienes hydroperoxides as a measure of lipid peroxidation levels.

Conjugated Dienes Hydroperoxides		STD			HFHS			HFHS + ω3	
		Mean		s.d. ^a^	Mean		s.d. ^a^	Mean	s.d. ^a^
DC (mmol Hydroperoxides/kg lipid)		15.82		0.49	23.32 *		2.15	21.85 *	2.48

^a^ s.d., standard deviation; * Pb.05 vs. STD control group.

**Table 6:** Carbonylated proteins identified in liver. Protein Spot No. refers to the numbered spots in 2-D gels shown in Figure 3. Green arrows indicate decrease in protein carbonylation. Red arrows indicate increase in protein carbonylation. The oxidative levels of other proteins were not affected by the dietary interventions. * Pb.05; ** Pb.01.

Spot No.	Identification	Gene Name	UniProtKB Code	Coverage (%)	Peptides	Unique	Avg. Mass	STD	HFHS	HFHS + ω3	HFHS/STD	HFHS + ω3/HFHS	Function
1	Carbamoyl-phosphate synthase [ammonia] mitochondrial	Cps1	P07756|CPSM	81	395	390	164,579	0.39 (0.20)	0.76 (0.10)	0.44 (0.06)	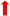 **	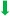 **	Urea cycle
2	Pyruvate carboxylase mitochondrial	Pc	P52873|PYC	40	63	63	129,777	1.01 (0.27)	0.99 (0.14)	0.61 (0.02)		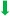 **	Gluconeogenesis Lipid biosynthesis Lipid metabolism
3	Dimethylglycine dehydrogenase mitochondrial	Dmgdh	Q63342|M2GD	61	100	100	96,047	0.44 (0.14)	0.67 (0.10)	0.60 (0.08)	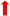 *		Amino-acid synthesis
4	Serum albumin	Alb	P02770|ALBU	83	149	149	68,731	0.88 (0.18)	1.49 (0.04)	0.91 (0.07)	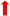 **	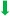 **	Protein metabolic and post-translational modification Fatty acids transport
5	Transketolase	Tkt	P50137|TKT	51	37	37	67,644	0.80 (0.21)	0.85 (0.12)	0.53 (0.08)		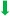 **	Glyceraldehyde-3-phosphate Biosynthetic process pentose-phosphate
6	Catalase	Cat	P04762|CATA	85	149	149	59,757	0.64 (0.16)	0.91 (0.01)	0.52 (0.00)	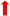 **	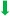 **	Hydrogen peroxide
7	Protein disulfide-isomerase A3	Pdia3	P11598|PDIA3	60	43	43	56,623	1.28 (0.19)	0.77 (0.20)	1.28 (0.03)	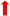 **	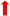 **	Cell redox homeostasis Protein folding
8	60 kDa heat shock protein mitochondrial	Hspd1	P63039|CH60	66	76	76	60,956	0.59 (0.15)	0.36 (0.03)	0.24 (0.06)	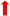 *	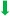 *	Host-virus interaction Protein folding
9	Glycerol kinase	Gk	Q63060|GLPK	47	29	26	57,477	0.62 (0.06)	0.53 (0.13)	0.32 (0.06)		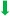 *	Glycerol metabolism Glycolysis
10	Formimidoyltransferase-cyclodeaminase	Ftcd	O88618|FTCD	48	35	35	58,914	0.54 (0.05)	0.43 (0.13)	0.29 (0.07)	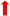 *		Histidine metabolism
11	Aldehyde dehydrogenase mitochondrial	Aldh2	P11884|ALDH2	82	135	120	56,488	0.59 (0.03)	0.85 (0.05)	0.65 (0.03)	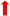 **	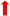 **	Alcohol metabolic process, Ethanol oxidation Carbohydrate metabolic process
12	Phenylalanine-4-hydroxylase	Pah	P04176|PH4H	54	40	39	51,822	1.23 (0.22)	1.19 (0.14)	0.82 (0.05)		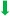 **	Amino-acid synthesis
13	Methylmalonate-semialdehyde dehydrogenase [acylating] mitochondrial	Aldh6a1	Q02253|MMSA	82	110	110	57,808	1.14 (0.58)	1.00 (0.45)	1.03 (0.08)			
	Dihydrolipoyl dehydrogenase mitochondrial	Dld	Q6P6R2|DLDH	67	50	50	54,038						
14	Alpha-aminoadipic semialdehyde dehydrogenase	Aldh7a1	Q64057|AL7A1	64	101	101	58,749	1.00 (0.08)	1.08 (0.21)	0.74 (0.02)		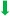 *	Cellular aldehyde metabolic Glutamate biosynthetic process Positive regulation of insulin secretion Tricarboxylic acid metabolic process
	Glutamate dehydrogenase 1 mitochondrial	Glud1	P10860|DHE3	76	150	150	61,416						
15	4-aminobutyrate aminotransferase mitochondrial	Abat	P50554|GABT	40	20	20	56,456	1.26 (0.46)	0.93 (0.28)	0.95 (0.38)			
16	Hydroxymethylglutaryl-CoA synthase mitochondrial	Hmgcs2	P22791|HMCS2	61	76	76	56,912	0.95 (0.37)	1.01 (0.41)	1.02 (0.12)			
17	6-phosphogluconate dehydrogenase decarboxylating	Pgd	P85968|6PGD	75	54	54	53,236	0.71 (0.11)	0.63 (0.13)	0.59 (0.17)			
18	Actin cytoplasmic 1	Actb	P60711|ACTB	85	104	10	41,737	1.15 (0.31)	1.19 (0.19)	0.67 (0.32)		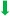 *	Cell motility Membrane organization
19	Isocitrate dehydrogenase [NADP] cytoplasmic	Idh1	P41562|IDHC	61	45	41	46,734	0.31 (0.16)	0.40 (0.16)	0.24 (0.04)			
	Beta-ureidopropionase	Upb1	Q03248|BUP1	51	54	54	44,042						
	Long-chain specific acyl-CoA dehydrogenase mitochondrial	Acadl	P15650|ACADL	69	67	67	47,873						
20	Fumarylacetoacetase	Fah	P25093|FAAA	68	64	64	45,976	0.48 (0.03)	0.75 (0.10)	0.50 (0.08)	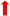 **	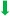 *	Amino-acid synthesis
21	Argininosuccinate synthase	Ass1	P09034|ASSY	76	101	101	46,496	1.00 (0.16)	1.87 (0.03)	0.90 (0.11)	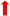 **	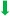 **	Urea cycle Amino-acid synthesis
22	Phosphoglycerate kinase 1	Pgk1	P16617|PGK1	77	62	25	44,538	0.52 (0.11)	0.89 (0.04)	0.59 (0.14)	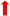 **	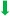 **	Glycolysis Fatty acid metabolism Lipid metabolism
	3-ketoacyl-CoA thiolase mitochondrial	Acaa2	P13437|THIM	69	33	33	41,871						
23	Alcohol dehydrogenase 1	Adh1	P06757|ADH1	80	123	117	39,645	1.09 (0.13)	1.16 (0.26)	0.59 (0.14)		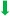 *	Ethanol oxidation Response to sex hormones Retinoid metabolic process
24	Fructose-bisphosphate aldolase B	Aldob	P00884|ALDOB	90	150	146	39,618	0.91 (0.06)	0.97 (0.04)	0.62 (0.08)	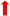 *	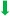 **	Glycolysis
25	3-oxo-5-beta-steroid 4-dehydrogenase	Akr1d1	P31210|AK1D1	77	54	50	37,378	0.38 (0.05)	0.32 (0.03)	0.19 (0.05)	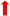 *	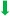 **	Bile acid catabolism Lipid metabolism Urea cycle
	Ornithine carbamoyltransferase mitochondrial	Otc	P00481|OTC	67	72	72	39,886						
26	Regucalcin	Rgn	Q03336|RGN	61	76	76	33,390	0.53 (0.11)	0.72 (0.19)	1.06 (0.27)	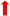 *	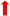 **	Ascorbate biosynthesis
27	Glyceraldehyde-3-phosphate dehydrogenase	Gapdh	P04797|G3P	53	32	10	35,828	0.59 (0.21)	0.55 (0.14)	0.75 (0.29)	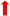 **		
28	Thiosulfate sulfurtransferase	Tst	P24329|THTR	50	41	41	33,407	0.50 (0.04)	0.57 (0.10)	0.63 (0.06)			
29	Malate dehydrogenase cytoplasmic	Mdh1	O88989|MDHC	66	45	45	36,483	0.41 (0.15)	0.77 (0.09)	0.41 (0.12)	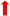 **	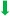 **	Tricarboxylic acid cycle
30	3-hydroxyanthranilate 3 4-dioxygenase	Haao	P46953|3HAO	45	21	21	32,582	0.72 (0.42)	0.81 (0.03)	0.51 (0.14)		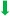 **	Pyridine nucleotide biosynthesis
31	Omega-amidase NIT2	Nit2	Q497B0|NIT2	33	11	11	30,701	1.89 (0.39)	1.95 (0.30)	1.31 (0.47)		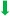 *	Amino-acid synthesis
32	Carbonic anhydrase 3	Ca3	P14141|CAH3	87	74	73	29,431	0.56 (0.12)	0.83 (0.17)	0.43 (0.09)	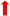 *	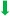 *	Response to oxidative stress
33	Glutathione S-transferase Mu 2	Gstm2	P08010|GSTM2	67	23	15	25,703	1.35 (0.37)	1.98 (0.12)	1.17 (0.31)	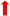 **	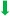 **	Cellular detoxification of nitrogen compound Glutathione metabolic process
34	Glutathione S-transferase Mu 2	Gstm2	P08010|GSTM2	84	62	43	25,703	0.95 (0.03)	1.35 (0.08)	0.93 (0.01)	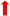 **	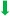 **	Cellular detoxification of nitrogen compound Glutathione metabolic process
35	Glutathione S-transferase Mu 2	Gstm2	P08010|GSTM2	72	28	18	25,703	0.72 (0.19)	2.25 (0.16)	1.14 (0.07)	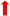 **	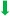 **	Cellular detoxification of nitrogen compound Glutathione metabolic process
36	3-hydroxyacyl-CoA dehydrogenase type-2	Hsd17b10	O70351|HCD2	80	38	38	27,246	0.56 (0.46)	0.98 (0.12)	0.62 (0.26)	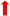 *	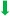 *	tRNA processing

## Supplementary Materials

The following are available online at http://www.mdpi.com/1660-3397/16/10/353/s1, Table S1: Diet composition, Table S2: Composition of the fatty acid diet.
