# Supplementary material for: Targeting Hepatic Protein Carbonylation and Oxidative Stress Occurring on Diet-Induced Metabolic Diseases through the Supplementation with Fish Oils
Source: Mar Drugs. 2018 Sep 26;16(10):353. doi: 10.3390/md16100353 (PMC6213247; doi:10.3390/md16100353)
Supplement: Supplementary file 1 [file marinedrugs-16-00353-s001.pdf]

Table S1: Diet composition. 27 male Sprague Dawley rats were randomly assigned to one of three dietary groups: a control standard diet group (STD) (n=9) fed a standard diet (Teklad Global 14% Protein Rodent Maintenance Diet, Harlan Laboratories, UK); a high-fat high-sucrose diet group (HFHS) (n=9) fed a high-caloric diet (TD.08811 45% kcal Fat Diet, Harlan Laboratories, UK) and a HFHS group supplemented with marine ω-3 PUFA (± 0.8mL/Kg body weight EPA/DHA 1:1 per week) for 9 weeks. Rats had *ad libitum* access to water and food.

Diet composition			
**Parameter**	**STD**	**HFHS**	**HFHS+ω3**
Flour (g)	1000[a]	1000[b]	1000[b]
Porcine gelatin (g)	25	25	25
Soybean lecithin (g)	6	22	22
Oil (mL)	19 mL soybean	24 mL soybean	24 mL EPA:DHA 1:1
**Macronutrients (% weight)**			
**Parameter**	**STD**	**HFHS**	**HFHS+ω3**
Protein	16.4	21.7	21.7
Fat	6.2	24.1	24.1
Carbohydrates	46.6	45.0	45.0
**Macronutrients (% caloric value)**			
**Parameter**	**STD**	**STD**	**HFHS+ω3**
Protein	21,3	17,9	17.9
Fat	18,2	44,9	44.9
Carbohydrates	60,5	37,2d	37.2d
Total energy density (kcal/g)	3.1	4.8	4.8

[a] Teklad Global 14% Protein Rodent Maintenance Diet, Harlan Laboratories, UK

[b] TD.08811 45% kcal Fat Diet, Harlan Laboratories, UK

Table S2: Composition of the fatty acid diet. Fatty acids composition of marine lipids enriched feed had higher proportions of EPA, DPA, DHA, and ARA than control supplements. Thereby, the fatty acids composition of control supplements had higher proportions of palmitic and oleic acids, linoleic acid, and alfa-linolenic acid than fish oil supplements. Fatty acid profile of control diet (% of total fat) was 17.5% saturated fatty acids (SFA), 21.1% monounsaturated fatty acids (MFA) and 61.1% polyunsaturated fatty acids (PUFA). In the HFHS diet, the fatty acid profile was 59.4% SFA, 29.3%MUFA and 10.8% PUFA. Results are expressed as a percentage of total fatty acids.

% FA	STD	HFHS	HFHS+ω3
14:0	0,017	11,5	11,6
16:0	14,8	32,2	32,0
16:1ω7	0,015	1,7	1,7
18:0	2,9	13,7	13,7
18:1ω9	20,5	26,4	26,2
18:1ω7	0,026	0,67	0,68
18:2ω6	58,6	9,7	8,7
20:0	n.d.	n.d.	0,0069
18:3ω3	2,9	1,1	1,1
20:1ω9	0,025	0,031	0,021
18:4ω3	0,0026	0,0032	0,033
20:2ω6	0,0034	0,0043	0,0045
20:3ω6	n.d.	n.d.	0,0048
20:4ω6	0,0069	0,0086	0,036
22:1ω11	0,019	0,023	0,024
22:1ω9	0,0043	0,0054	0,006
20:4ω3	0,0034	0,0043	0,022
20:5ω3	0,012	0,015	0,54
24:1ω9	0,0048	0,0060	0,0082
22:5ω3	0,0045	0,0056	0,093
22:6ω3	0,02	0,024	0,56
Total SFAs	17,5	59,4	60,5
Total MUFAs	21,1	29,3	28,4
Total PUFAs	61,1	10,8	10,7
Total ω3	3,3	1,6	1,4
Total ω6	58,8	10,4	10,6
